# Effect of modified gastrointestinal decompression under abdominal CT in patients with intestinal obstruction

**DOI:** 10.1308/rcsann.2023.0073

**Published:** 2023-11-20

**Authors:** X Wu, M Chen, X Yu, B Wu

**Affiliations:** Ningde Municipal Hospital of Ningde Normal University, China

**Keywords:** Gastrointestinal decompression, Catheter method, Abdominal computerized tomography scan

## Abstract

**Introduction:**

We aimed to evaluate the effect of continuous quality improvement on modified gastrointestinal decompression under abdominal computed tomography (CT) in patients with intestinal obstruction.

**Methods:**

The CT images of 74 patients with intestinal obstruction who underwent gastrointestinal decompression in our hospital from 1 January 2018 to 31 December 2019 were analysed retrospectively (Control group). Factors influencing unsatisfactory decompression effects were analysed, and corresponding improvement measures were formulated and implemented. A total of 77 patients from 1 January 2020 to 31 March 2022 were enrolled prospectively (Study group). The position of the nasogastric tube end, the amount of gastric drainage within 24h and the degree of abdominal distension relief were compared before and after the improvement.

**Results:**

After implementation of continuous quality improvement, the proportion of the end of the nasogastric tube reaching the antrum, the amount of gastric fluid drainage within 24h and the degree of abdominal distension relief were better than those before improvement (*p*<0.001, respectively). The execution rate and accuracy rate of CT interpretations by nurses reached 100% and 82%, respectively.

**Conclusions:**

Modified gastrointestinal decompression based on abdominal CT scans can increase the success of gastrointestinal decompression and effectively reduce the discomfort of patients.

## Introduction

Acute intestinal obstruction occurs when the forward flow of intestinal contents is interrupted or impaired by a mechanical cause. Imaging with ultrasound, abdominal radiography or computed tomography (CT) can confirm the diagnosis and assist in decision making for therapeutic planning. With the development of medical imaging, an abdominal CT scan has become a common and reliable method to judge the condition of patients with intestinal obstruction.^1,2^ A CT scan combines a series of x-ray images taken from different angles to produce cross-sectional images. These images are more detailed than a standard x-ray, and are more likely to show an intestinal obstruction. An abdominal CT scan can present the gastrointestinal tract clearly and intuitively, including the retention of gastric contents and the shape and end position of the gastrointestinal decompression tube in the stomach.^2^

Treatment is fluid resuscitation, nasogastric suction and, in most cases of complete obstruction, surgery. Patients with partial obstruction with no sign of strangulation should initially be treated conservatively. Bologna guidelines pointed out that nonoperative treatment, especially nasogastric or long-tube decompression, is effective in most patients.^3^ Effective gastrointestinal decompression can discharge the fluid and gas in the gastrointestinal tract, reduce the gastrointestinal pressure, relieve abdominal distension, improve the blood circulation of the gastrointestinal wall and promote the recovery of gastrointestinal function.

Over the past 30 years, image-guided placement of gastrostomies and cecostomies for gastrointestinal decompression has developed into a safe and effective treatment for symptomatic bowel obstruction. This study aimed to evaluate whether the improved method of gastrointestinal decompression based on CT scan results can improve the effect of gastrointestinal decompression.

## Methods

### Patients

From 1 January 2018 to 31 March 2022, a total of 415 patients were diagnosed with intestinal obstruction. After screening and selection, the data of 74 patients from 1 January 2018 to 31 December 2019 were collected and analysed retrospectively (Control group). From 1 January 2020 to 31 March 2022, a total of 77 cases were recruited prospectively with the improved method of gastrointestinal decompression and catheterisation (Study group).

Patients were included if (1) they met the diagnostic criteria of intestinal obstruction;^2^ (2) the gastrointestinal decompression was performed for more than 24h; (3) abdominal CT scan was performed during gastrointestinal decompression. Patients were excluded if (1) there was difficulty placing a gastric tube, such as tracheal intubation, tracheotomy, no pharyngeal reflex, coma or elderly patients; (2) they received gastrectomy because of stomach disease; (3) the obstruction-related surgery was performed on the day of catheterisation.

All patients had been informed and signed consent. This study was approved by Ethics Committee of our hospital (approval ID: 20220903).

### Methods

A gastrointestinal decompression catheterisation continuous improvement group in the general surgery department was set up, including one chief physician, two attending physicians, one attending physician of the imaging department, two co-chief superintendent nurses and seven supervisor nurses. The doctors supplied technical support in image interpretation. The head nurse reviewed the literature, formulated improvement measures, regularly collated the feedback improvement effect and existing problems and organised discussion and analysis to put forward further rectification. Nurses collected and analysed the clinical data, assisted in the implementation of improvement measures, organised learning-related content and followed up medical records.

The abdominal CT results of 74 patients with intestinal obstruction who underwent gastrointestinal decompression from 1 January 2018 to 31 December 2019 were collected and analysed, including the situation of gastric tube in the stomach, gastric retention and the depth of gastric tube insertion. The gastric tube is not straight in the descending process; therefore, what we found on CT is that there are roughly two types of gastric tube morphology in the stomach. One is to continue to follow the second curve to reach the antrum region or nearby, and the other is to follow the first physiological curve to the lower left region (gastric body) or to the cardiac region.

When the patient is supine, the fluid level of the gastric contents can reach the vicinity of the gastric cardia. Patients are often required to adopt a supine position with an indwelling silicone gastric tube (Type and Specification: 4.7mm/F14, Yangzhou New Star Silicone Factory).

Although the end of the gastric tube reaches only the cardia or upper part of the stomach body after catheterisation according to the previous method of measuring depth, there still will be fluid elicitation. However, when the patient was in semi-supine position or standing, the fluid level dropped away from the gastric cardia, resulting in no longer drawing out gastric fluid, which was consistent with the analysis of Yang *et al*.^4^ According to the interview with general practice nurses, in daily work, doctors will ask nurses to increase the depth of catheterisation when they judge that drainage is not enough according to CT results and the degree of abdominal distension relief.

After years of experience with gastrointestinal decompression and catheterisation, nurses from the surgical department will insert longer gastric tubes after the scale specified in the textbook, but each person may increase the depth of insertion without establishing a specification.^5^ Based on our previous experience, we formulated the following improvement measures:

(1) Combined with the imaging data, the problems of 74 patients with gastrointestinal decompression were reported to team members to determine a rectification target.
(2) Doctors and nurses in the department were organised to learn and master the skills of abdominal CT scan interpretation, such as the situation of gastric content retention and the position and shape of the gastric tube. With the support of the network information department, a picture archiving and communication system was installed on all nursing computers, and permissions were opened to facilitate the operation and reference of clinical nurses.
(3) The depth of gastrointestinal decompression catheterisation was increased to 10cm from hairline to the xiphoid process. The position of catheterisation was adjusted to a high slope recumbent position, and the head of the bed elevated as far as possible (>45°).
(4) Abdominal CT scan was included in the evaluation to determine the shape of the stomach, evaluate whether the stomach contents were solid or liquid, identify the shape of the gastric tube and the location of the end, compare the consistency between the gastric retention and the amount of gastric drainage, and analyse the reasons for timely adjustment to ensure effective decompression if there was any discrepancy.

The primary nurse filled in the corresponding form, including the patient's name, bed number, gastric tube placement time, depth, whether there is adjustment and adjustment depth, etc. The rest of the operation process and nursing measures were carried out as usual.

Quality control inspector (QCI) nurses checked whether the daily data registration was complete, asked whether there were any special circumstances during catheterisation, and checked the performance of interpretation of the CT by the primary nurse. The same patient had an abdominal CT scan before and after catheterisation. The QCI nurse and the surgeon jointly checked the interpretation accuracy of the nurses, and one error was considered inaccurate. QCI nurses also need to follow up cases and collect data. The implementation rate, accuracy rate and improvement effect were summarised monthly. In addition, sources of problems in the implementation process were analysed and improvement measures were put forward.

### Evaluation indicator

Position of the end of the gastric tube: to facilitate description of the end of the tube, we divided the stomach into the cardia of the stomach, the upper gastric body, the lower gastric body and the antrum region.

Drainage effect: drainage volume and degree of abdominal distension in the first 24h.

The degree of abdominal distension was graded as follows^6^: no abdominal distension scored one point, abdominal distension but abdominal tenderness scored two points, obvious abdominal distension without abdominal pain scored three points and abdominal distension with abdominal pain scored four points.

### Statistical analysis

All statistical analyses were performed using IBM SPSS Statistics 23.0 software (IBM SPSS Statistics). The measurement data were tested for normality. Data conforming to normal distribution were expressed as mean±standard deviation, and data not conforming to normal distribution were expressed as median and interquartile. Count data were expressed as frequency and percentage. Data were compared using Student's *t* tests, Fisher's exact tests or Chi-square tests, as appropriate. *p*<0.05 was considered significant difference.

## Results

### Baseline characteristics

A total of 74 hospitalised patients (49 male, 25 female) from 1 January 2018 to 31 December 2019 were selected as the control group according to the continuous improvement time. Patient age ranged from 34 to 88 years (62.0±11.5 years). A total of 77 patients (52 male, 25 female) from 1 January 2020 to 31 March 2022 were selected as the study group. Patient age ranged from 31 to 78 years (60.5±10.9 years). There was no significant difference in age, gender, height and degree of abdominal distension between the two groups (*p*>0.05).

In the study group, there were three cases of gastric tube retraction in the stomach due to excessive food residue in the stomach. Gastric drainage was insufficient; thus, these three cases were eliminated.

### Analysis of control group

**Figure 1:**
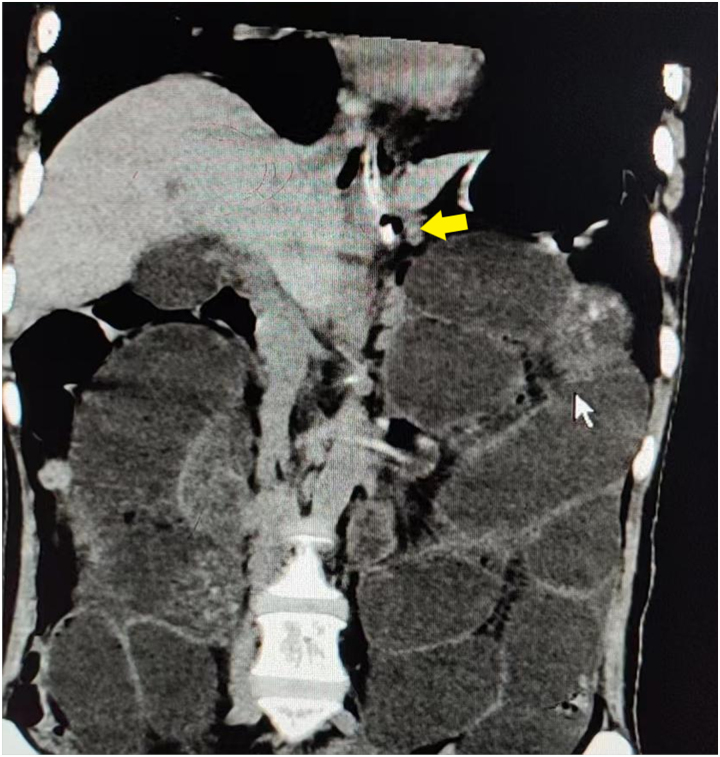
CT scan of a typical case: the position of the nasogastric tube end stayed in the gastric cardiac region (yellow arrow). CT = computed tomography

**Figure 2:**
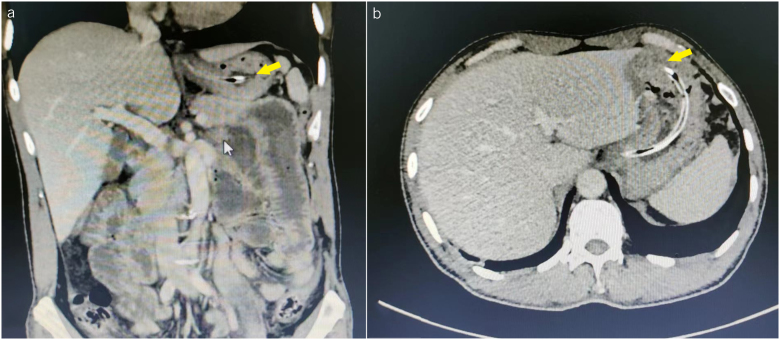
CT scan of another typical case showed the position of the nasogastric tube end in the gastric fundus: (a) coronal section: after entering the gastric cardia, the nasogastric tube was oriented to the left (yellow arrow); (b) cross-section: the nasogastric tube was horizontal after entering the gastric cardia (yellow arrow). CT = computed tomography

By analysing the effect of gastrointestinal decompression in the control group, the following problems were detected: (1) the end of the gastric tube did not reach the antrum region in 65 cases, including 23 cases in the cardiac region (Figures 1 and 2), 28 cases in the upper part of the stomach body and 14 cases in the lower part of the stomach body. (2) There were 42 patients with obvious gastric distension but less than 200ml gastric drainage volume in the first 24h after catheterisation. Among them, four cases were less than 100ml. (3) Among these patients, there was no correlation between patient height and the depth of the gastric tube. For example, the catheterisation depth of patients with the same height of 160cm ranged from 50cm to 60cm.

### Comparison of the position of gastric tube end

**Figure 3:**
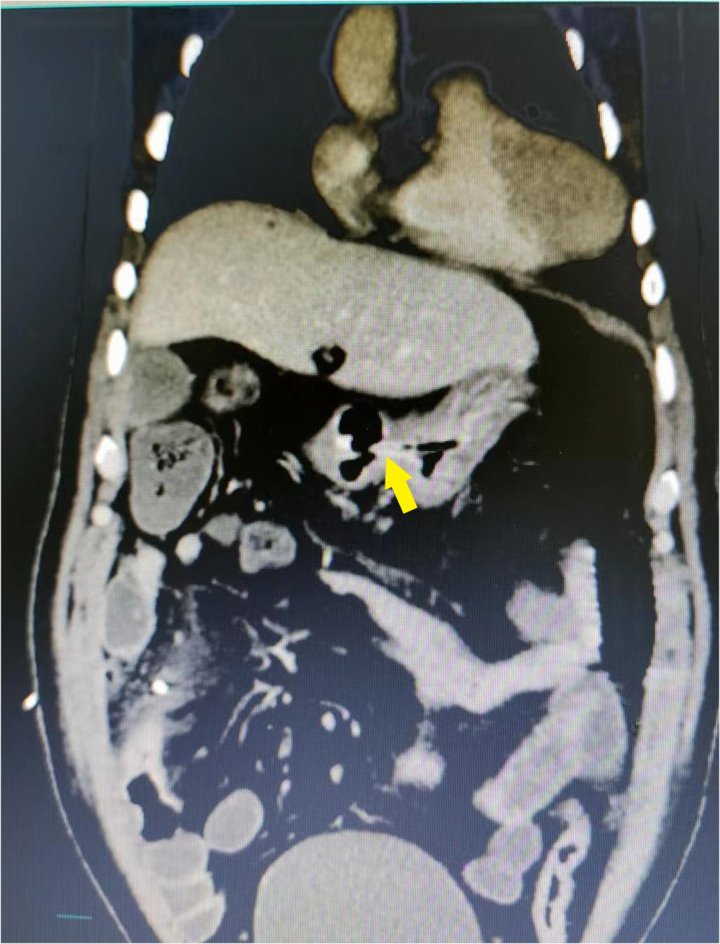
CT scan of a ideal case showed the nasogastric tube end reached the antrum of stomach (yellow arrow). CT = computed tomography

As the study group increased the depth of the gastric tube insertion, none of the tube ends were found in the cardiac region, whereas there were 23 such cases in the control group. The number of cases where the position of tube ended in upper gastric body, lower gastric body and antrum region (Figure 3) were 21, 29 and 24, respectively. Correspondingly, the control group had 28, 14 and 9 cases in the above position (Table 1). There was significant difference in the position of gastric tube end before and after improvement (*p*<0.05).

**Table 1:** Comparison of position of end of gastric tube before and after improvement

Sites	Study group (*n*=74)	Control group (*n*=74)	*χ* ^2^	*p*-value
Cardiac region	0	23	36.051	<0.001
Upper gastric body	21	28		
Lower gastric body	29	14		
Antrum region	24	9		

### Comparison of height, depth of gastric tube and 24h drainage volume

There was no statistical difference in height between the two groups (*p*>0.05), but the depth of the gastric tube was significantly deeper in the study group than in the control group (58.22±3.53 vs 52.39±3.90, *p*<0.01), and 24h drainage volume was greater in the study group (322.84±112.99 vs 190.49±38.31, *p*<0.01) (Table 2).

**Table 2:** Comparison of height, depth of gastric tube and 24h drainage volume

Items	Study group (*n*=74)	Control group (*n*=74)	*t* value	*p*-value
Height (cm)	163.82±6.79	163.77±6.83	0.039	0.969
Depth of gastric tube placement (cm)	58.22±3.53	52.39±3.90	7.651	<0.001
24h drainage volume (ml)	322.84±112.99	190.49±38.31	7.395	<0.001

### Comparison of degree of abdominal distension

Before gastric intubation, the degree of abdominal distension between the two groups was comparable (*p*=0.582). After gastric intubation, patients in both groups were significantly relieved, but efficacy of relief in the study group was better (*p*=0.006) (Table 3).

**Table 3:** Comparison of abdominal distension degree

Abdominal distension score		Study group (*n*=74)	Control group (*n*=74)	*χ* ^2^	*p*-value
Before gastrointestinal decompression	≤2	22	19	0.304	0.582
	>2	52	55		
After gastrointestinal decompression	≤2	61	46	7.591	0.006
	>2	13	28		

## Discussion

The results of this study showed that, after continuously improving the method of gastrointestinal decompression, the end of the gastric tube can reach the antrum to a greater extent, with greater drainage and better effect on relieving abdominal distension.

We speculate that the reasons for the problems mentioned in the results section are as follows: (1) anatomically, the xiphoid process is located roughly at the same level as the end of the oesophagus. According to the textbook measurement method, i.e. the depth of catheter placement for gastrointestinal decompression is from hairline to xiphoid process, the end of the tube will reach only the end of the oesophagus or the stomach cardia, which is consistent with the results of Li *et al*.^6^ (2) By interpreting the CT, we also found that the stomach was elevated due to intestinal distension and compression. When the gastric tube reaches the antrum region from the oesophagus, it needs to go through two physiological curves. The first is the oesophagocardiac junction that runs to the lower left, and the second is the stomach structure that runs to the lower right.

The gastric tube used in this study was made of silicone (Yangzhou Xinxing Silica Gel Factory, Jiangsu, China), and the size used was F14 in all cases. The length of the gastric tube was 100cm, without guide wire, and it is relatively soft. Our in vitro experiments showed that bending could be reduced by gravity, thus the position of catheterisation was determined to be a high slope recumbent position due to retraction.

The tube has three lateral holes, with the last hole 10cm from the end. Considering that the three lateral holes of the gastric tube had reached the stomach after increasing the depth by 10cm, in order to avoid excessive insertion into the duodenum, which would affect the drainage effect, we took an increase of 10cm as the benchmark and made adjustments according to the actual situation.

In the study group, there was no case that needed to increase the depth of intubation. Five patients had obvious gastric drainage during the intubation process. When the intubation was continued to the predetermined scale, drainage was inadequate, and it was withdrawn to the position of smooth drainage. The height of these patients was 143–150cm, and the final depth increase was 6–8cm. In addition, it can be seen from the results in Table 2 that gastric tube insertion depth was increased by about 5cm rather than 10cm on average compared with that before the improvement, which is consistent with the interview results of nurses in the previous status quo cause analysis.

Through this improvement, we developed new procedures and norms for gastrointestinal decompression and catheterisation to ensure effective drainage. The gastrointestinal drainage volume reached 322.84ml within 24h, which was significantly higher than 190.49ml before the improvement and had statistical significance. The degree of abdominal distension was relieved in both groups, but the rate of remission after improvement was also greater than that before improvement.

Previous studies indicated that increasing the depth of nasogastric tube insertion on the basis of traditional nasogastric tube insertion can improve the effect of gastrointestinal decompression, but there is no uniform standard on how much depth to increase.^4–8^

Some studies determined the precise insertion depth of nasogastric tube intubation according to the judgment of doctors during the operation or using a scale under gastroscope,^9,10^ but the actual shape of the nasogastric tube in the stomach and individual differences were not taken into consideration. In addition to traditional methods, many new methods have been reported, such as x-ray,^11–13^ ultrasonography,^14^ pH offer,^12,13^ CO_2_ test^12,13^ or electromagnetic (EM) trace.^12^ EM trace and x-ray can accurately determine the specific position of the gastric tube, but they increase the risk of nasogastric tube position displacement and radiation accumulation.^12^ In addition, it is difficult for clinical nurses to implement the above measures due to busy clinical work, limited economic conditions of patients, lack of support from managers and inadequate cooperation from medical and technical departments.

Based on a routine CT scan of patients with intestinal obstruction, this study made full use of the advantages of abdominal CT three-dimensional imaging to improve the procedure. The results of imaging examination were applied in the procedures, and the reasons for the unsatisfactory gastrointestinal decompression were explained in detail to the nurses. Through CT results, nurses can see whether the indwelling gastric tube is appropriate and the effect of gastrointestinal decompression, which stimulates their interest in exploring improvement. Through this improvement, the execution rate and accuracy rate of imaging interpretations by nurses reached 100% and 82%, respectively, which also realised an expansion of specialised nursing skills of the department. In this study, we formulated a modified gastrointestinal decompression based on abdominal CT scans, which provided insights for clinicians to alter their practice with patients with intestinal obstruction.

This study also has some limitations. First, this was not a randomised controlled trial, and the two groups of patients were not compared at the same time period, which may have some bias. Secondly, due to the limitation of the number of patients, sample size was not calculated in this study.

## Conclusion

Modified gastrointestinal decompression based on abdominal CT scans can increase the success of gastrointestinal decompression and effectively reduce the discomfort of patients.
